# Asymptomatic WPW Pattern Detected by School ECG Screening: Prevalence, Phenotype, and Automated Interpretation Errors

**DOI:** 10.3390/biomedicines14040807

**Published:** 2026-04-02

**Authors:** Jano Mathias Kosing, Lucian Mureşan, Gabriel Gusetu, Radu Rosu, Dana Pop, Cecilia Lazea, Simona Sorana Căinap, Alina Negru, Gabriel Cismaru

**Affiliations:** 1 Cardiology, Rehabilitation, “Iuliu Hatieganu” University of Medicine and Pharmacy, 400000 Cluj-Napoca, Romania; janokosing@gmail.com; 2 Cardiology Department, Emile Muller Hospital, 68100 Mulhouse, France; lmure_san@yahoo.com; 3 Fourth Department of Internal Medicine, Cardiology-Rehabilitation, “Iuliu Hatieganu” University of Medicine and Pharmacy, 400015 Cluj-Napoca, Romania; gusetu@gmail.com (G.G.); rosu.radu1053@gmail.com (R.R.); pop67dana@gmail.com (D.P.); 4 Department of Pediatrics I, Emergency Clinic Hospital for Children, “Iuliu Hatieganu” University of Medicine and Pharmacy, 400012 Cluj-Napoca, Romania; cicilazearo@yahoo.com; 5 Department of Pediatrics II, Emergency Clinic Hospital for Children, “Iuliu Hatieganu” University of Medicine and Pharmacy, 400012 Cluj-Napoca, Romania; cainap.simona@gmail.com; 6 Department of Cardiology II, University of Medicine and Pharmacy “Victor Babes”, 300041 Timisoara, Romania; eivanica@yahoo.com; 7 Rocordis Heart Center, Institute of Cardiovascular Diseases Timișoara, 300041 Timisoara, Romania

**Keywords:** Wolff–Parkinson–White, ventricular preexcitation, prevalence, screening, children, electrocardiography, automated ECG interpretation

## Abstract

**Background/Objectives:** The Wolff–Parkinson–White (WPW) pattern is characterized by ventricular preexcitation due to an accessory atrioventricular pathway. Population-based data on the prevalence of asymptomatic WPW patterns in children are limited, and automated ECG interpretation may be misleading in the setting of preexcitation. Our aim was to determine the prevalence of the WPW pattern in a large cohort of asymptomatic Romanian school children and to describe electrocardiographic characteristics, ECG-based accessory pathway localization, and automated ECG interpretation errors. **Methods:** We performed a retrospective cross-sectional analysis of 12-lead ECGs obtained during a school-based screening program in Romania (May–December 2015). After exclusion of duplicates, technical errors, and participants outside the prespecified age range, 24,112 unique children aged 6–18 years were included. The WPW pattern was adjudicated by pediatric electrophysiologists. Prevalence was estimated using the Wilson score method. Sex differences were assessed using Fisher’s exact test. **Results:** The WPW pattern was identified in 18/24,112 children, yielding a prevalence of 0.075% (0.75 per 1000). The WPW pattern was more frequent in boys than girls (12/11,858 (0.10%) vs. 6/12,255 (0.048%), *p* = 0.18). Most cases demonstrated mild preexcitation, with only a minority showing marked QRS widening. ECG-based algorithms suggested a predominance of left-sided accessory pathways. Automated ECG interpretation frequently produced misleading diagnostic statements, including bundle branch block/intraventricular conduction delay (5/18; 27.8%) and pseudo-infarction/ischemia patterns (1/18; 5.6%), and did not explicitly identify WPW/preexcitation. **Conclusions:** In a large school-based screening cohort of asymptomatic Romanian children, WPW pattern prevalence was 0.074%, with a trend toward male predominance. Most cases exhibited mild preexcitation. Automated ECG interpretation commonly misclassified preexcitation-related ECG findings, highlighting the importance of expert ECG review in pediatric screening programs.

## 1. Introduction

The Wolff–Parkinson–White (WPW) pattern is characterized by ventricular preexcitation due to the presence of an accessory atrioventricular pathway, resulting in early activation of the ventricles. With surface electrocardiogram (ECG), the WPW is classically identified by a short PR interval, a delta wave reflecting slurred initial ventricular depolarization, and varying degrees of QRS widening [1,2,3].

Although many individuals with the WPW pattern remain asymptomatic, its clinical relevance lies in the potential to facilitate atrioventricular re-entrant tachycardia and, in rare cases, rapid conduction of atrial fibrillation over the accessory pathway, which may lead to life-threatening ventricular arrhythmias [4,5,6]. This risk is of particular concern in pediatric populations, where the WPW pattern is often detected incidentally and where risk-stratification strategies remain a subject of ongoing clinical debate.

In children, the WPW pattern is most commonly identified during routine or targeted screening, including school-based medical evaluations, pre-participation sports assessments, or incidental ECG recordings [7,8,9]. However, the detection of ventricular preexcitation in this population presents specific challenges. Pediatric ECGs often demonstrate subtle or intermittent preexcitation due to rapid atrioventricular nodal conduction and fusion of activation, which may result in less pronounced PR shortening and QRS widening compared with adults [10,11,12].

An additional challenge in contemporary screening practice is the widespread use of automated ECG interpretation systems. While these algorithms are useful for high-throughput analysis, they are prone to misclassification in the presence of preexcitation, frequently generating misleading diagnostic statements such as bundle branch block, intraventricular conduction delay, ventricular hypertrophy, or pseudo-ischemic patterns [13,14,15,16]. Such inaccuracies may lead to inappropriate reassurance or unnecessary downstream investigations, particularly in large-scale screening settings.

Despite the clinical and public health relevance of the WPW pattern, population-based data on its prevalence in children remain limited, particularly in Eastern European populations. Reported prevalence varies across studies, reflecting differences in screening methodology, age distribution, and diagnostic criteria, as well as the extent of expert ECG adjudication [7,8]. In Romania, available data are sparse and largely derived from clinical or electrophysiology-based cohorts, with a lack of large-scale screening studies providing robust prevalence estimates in asymptomatic children.

Establishing accurate prevalence data in pediatric populations is important for informing screening strategies, resource allocation, and downstream clinical pathways, including referral and risk stratification. In addition, better characterization of ECG phenotypes and potential pitfalls in automated interpretation may improve diagnostic accuracy in real-world screening programs.

Therefore, the aim of the present study was to determine the prevalence of the WPW pattern in a large cohort of asymptomatic Romanian school children aged 6–18 years who underwent ECG screening. Secondary objectives were to describe electrocardiographic characteristics of the WPW pattern, apply ECG-based algorithms for accessory pathway localization, and evaluate the frequency and nature of automated ECG interpretation errors in this population [4,17,18]. 

## 2. Materials and Methods

### 2.1. Study Population

This study was a retrospective, observational, cross-sectional analysis of electrocardiographic screening data collected in school-aged children. Between May 2015 and December 2015, a total of 24,316 children were included in the Cardioped prevalence study, where 12-lead ECGs were recorded from students attending 12 schools in northwest Romania (Bihor, Bistrița-Năsăud, Cluj, Satu Mare, Sălaj, Maramureș) and west Romania (Arad, Hunedoara, Timiș, and Caraș-Severin). Only asymptomatic children without known cardiovascular disease were eligible for inclusion. Children with known cardiac disease or symptoms suggestive of cardiac disease were excluded. None of the included participants were receiving cardiac medication at the time of ECG acquisition. For children recorded multiple times, only one ECG recording per participant was retained. Participants were categorized into the following age strata (based on completed years of life): 6 years, 7–10 years, 11–14 years, and 15–18 years. Ethical approval was obtained from the Ethics Committee of the “Iuliu Hațieganu” University of Medicine and Pharmacy. Administrative agreement was obtained from participating schools. Written informed consent was obtained from parents or legal guardians prior to inclusion.

### 2.2. ECG Acquisition and Interpretation Workflow

All children underwent a standard 12-lead digital ECG recording using BTL-08 MT Plus devices (BTL Industries, Stevenage, Hertfordshire, United Kingdom). ECGs were recorded at a sampling rate of 2000 Hz; the frequency response of the recorder was flat up to 170 Hz. ECGs were initially reviewed by approximately 200 physicians (cardiologists and pediatricians) over a 3-month period. Cases were flagged as suspected ventricular preexcitation based on the presence of electrocardiographic features suggestive of the WPW pattern, including short PR interval, delta wave morphology with slurred upstroke of the QRS complex, and QRS widening, while acknowledging that these features may be subtle in pediatric populations. All ECGs flagged as suspicious were subsequently subjected to expert adjudication by three independent pediatric electrophysiologists, who confirmed or excluded the diagnosis of the WPW pattern. Final-case classification in the present study is based exclusively on this expert consensus review. The use of expert adjudication by 3 electrophysiologists ensures a high level of diagnostic consistency.

All ECG recordings were obtained using the same device (BTL-08 MT Plus, BTL Industries), which incorporates a manufacturer-specific automated ECG interpretation system integrated within the BTL CardioPoint software. This system uses signal preprocessing, feature extraction (including PR, QRS, QT intervals, and waveform morphology), and a rule-based pattern recognition approach. Thus, all ECGs in the study were analyzed using a uniform, device-integrated software system.

Automated interpretation outputs were available for the entire screened cohort. However, the analysis of automated ECG interpretation errors was specifically performed in the subset of ECGs with confirmed WPW pattern, as determined by pediatric electrophysiologists. In this subgroup, automated interpretations were systematically reviewed and classified into categories, including false-positive diagnostic statements and failure to recognize ventricular preexcitation.

The WPW pattern was defined on surface ECG by the presence of ventricular preexcitation consistent with an accessory atrioventricular pathway, including a short PR interval (typically <120 ms), slurred upstroke of the QRS complex (delta wave), and QRS prolongation (classically >120 ms), acknowledging that QRS duration may remain near-normal in children due to fusion and rapid AV nodal conduction. 

ECG-based localization of accessory pathways was performed using the Arruda algorithm.

To estimate the nationwide burden of asymptomatic WPW pattern among Romanian children aged 6–18 years, demographic data from the Institutul Național de Statistică (INS) were used, based on the usually resident population on 1 July 2015, corresponding closely to the screening period. Observed prevalence estimates were extrapolated to this population to derive an estimated number of children with WPW pattern.

### 2.3. Statistics

Categorical variables (e.g., WPW pattern, sex, age strata) are presented as counts (n) and percentages. Continuous variables are presented as mean ± standard deviation (SD) or median with interquartile range (IQR), depending on distribution. Normality of continuous variables was assessed graphically (histograms and Q–Q plots) and tested using the D’Agostino–Pearson test, selected due to the large sample size. The prevalence of the WPW pattern was calculated using the Wilson score method. Associations between categorical variables were assessed using Fisher’s exact test. A two-sided *p*-value of <0.05 was considered statistically significant. Statistical analysis was performed using SPSS version 24.

## 3. Results

A total of 24,316 12-lead ECG recordings were collected during the school-based screening program conducted between May and December 2015. After the application of predefined exclusion criteria, including duplicate recordings, missing identification data, missing recording dates, and technical recording errors, as well as participants outside the prespecified age interval (age > 18 years, *n* = 75; age < 5 years, *n* = 2), a total of 24,112 unique children were included in the final analysis. The cohort consisted of 12,255 girls (50.8%) and 11,858 boys (49.2%) aged 6 to 18 years. The baseline characteristics of the study population, including age distribution across predefined strata, are presented in Table 1.

Among the 24,112 screened children, 18 ECGs fulfilled criteria for the WPW pattern, corresponding to an overall prevalence of 0.075% (0.75 per 1000 children; 7.5 per 10,000 children), 95% CI (0.047–0.118). Prevalence estimates were calculated using the Wilson score method. The WPW pattern was observed in 12/11,858 boys (0.10%) and 6/12,255 girls (0.048%). WPW prevalence was higher in males than in females; however, this difference did not reach statistical significance (Fisher’s exact test *p* = 0.18). Detailed sex-specific prevalence estimates are shown in Table 2. 

Age-stratified WPW prevalence is summarized in Table 3. The highest prevalence was observed among 6-year-old children, followed by the 11–14 years and 15–18 years age groups, whereas the lowest prevalence was observed in the 7–10 years group. 

According to data from the Institutul Național de Statistică (INS), the total resident population of Romanian children aged 6 to 18 years on 1 July 2015 was 2,990,843, including 1,535,588 males (51.3%) and 1,455,255 females (48.7%). Extrapolating the observed WPW pattern prevalence of 0.074% to this national population yields an estimated 2213 children with an asymptomatic WPW pattern on ECG. This estimate assumes that the screened cohort is broadly representative of the Romanian pediatric population aged 6–18 years. However, it should be interpreted cautiously, as the sample was derived from selected regions and included only asymptomatic children without known cardiovascular disease.

In the WPW cohort (*n* = 18), the median heart rate was 87 bpm (IQR 81–95.5). The median QRS duration was 90 ms (IQR 90–100), the median QT interval was 350 ms (IQR 338–370.5), and the median QTc was 420 ms (IQR 413.5–423.5). The median QRS axis was 45° (IQR 20–62.5).

Accessory pathway localization was assessed using the ECG-based Arruda algorithm. Based on surface ECG criteria, accessory pathways were predominantly left-sided (~67%), with the remaining pathways distributed equally between right-sided and septal locations (~17% each). Localization accuracy was limited in cases with minimal preexcitation.

**Figure 1:**
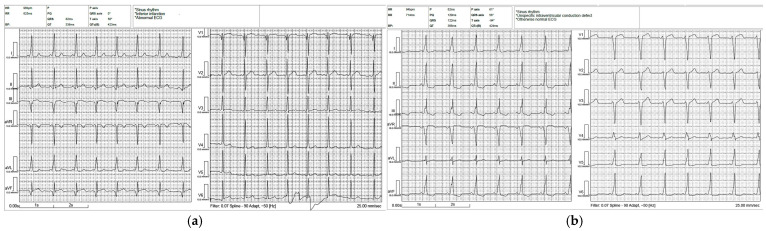
Mild preexcitation compared to strong preexcitation. (**a**) The image shows mild preexcitation, and the automated ECG interpretation states inferior myocardial infarction. (**b**) The image shows strong preexcitation, and the automated ECG interpretation states an unspecific intraventricular conduction defect.

Because PR interval and QRS duration may remain near-normal in children due to rapid atrioventricular nodal conduction and fusion, the degree of preexcitation was graded primarily based on the extent of accessory pathway contribution to ventricular activation. Preexcitation severity was classified as follows: strong preexcitation (*n* = 2)—prominent delta waves in multiple leads with clearly widened QRS complexes (typically ≥120 ms); moderate preexcitation (*n* = 9)—definite delta waves with mildly prolonged QRS duration (~100–119 ms); mild preexcitation (*n* = 7)—subtle delta waves/minimal slurring with QRS duration usually < 100 ms (Figure 1).

**Figure 2:**
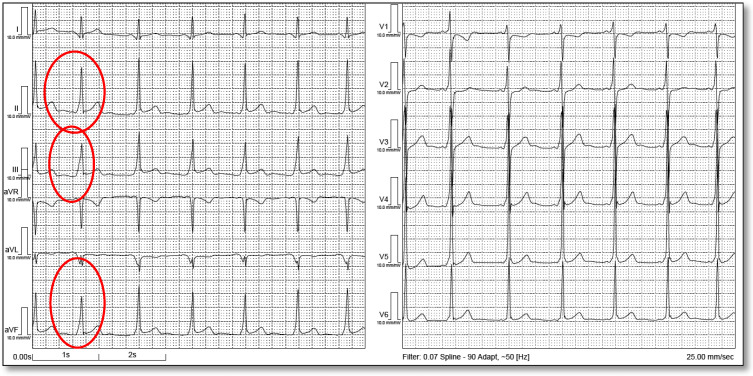
The image shows WPW pattern with early repolarization pattern in the inferior leads.

**Figure 3:**
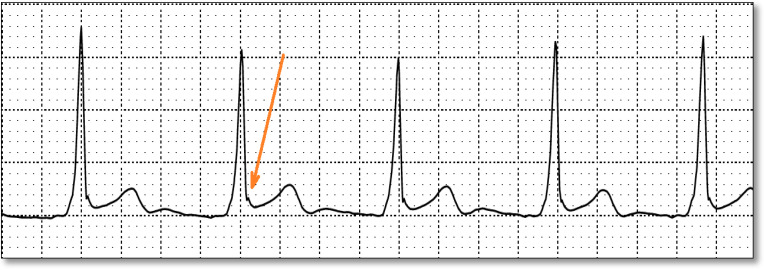
The image shows notched transition at the end of QRS into the ST segment.

Among the 18 ECGs, 1 ECG demonstrated features consistent with an early repolarization pattern, including J-point elevation above baseline in leads II, III, and aVF; concave ST-segment morphology; and a notched/slurred transition at the end of the QRS complex into the ST segment (Figure 2 and Figure 3).

### Automated ECG Interpretation Errors

Automated ECG interpretation algorithms are known to have limited reliability in pediatric populations and in the setting of ventricular preexcitation. In this cohort, automated interpretation statements frequently suggest alternative diagnoses related to secondary preexcitation-associated conduction and repolarization changes. Specifically, the ECG software flagged infarction/ischemia patterns in 1/18 (5.6%), bundle branch block or nonspecific intraventricular conduction delay (BBB/IVCD) in 5/18 (27.8%), and axis deviation (left or right) in 3/18 (16.7%) (Table 4).

Notably, none of the automated interpretation statements explicitly identified the WPW pattern, despite expert adjudication confirming preexcitation in all 18 ECGs.

## 4. Discussion

This large school-based ECG screening study provides contemporary prevalence estimates for the WPW pattern among asymptomatic Romanian children aged 6–18 years and highlights important electrocardiographic and interpretative characteristics relevant to pediatric cardiology practice.

### 4.1. Prevalence of Asymptomatic WPW Pattern

In this cohort of 24,112 asymptomatic children, the WPW pattern was identified in 18 individuals, corresponding to a prevalence of 0.074%. This screening-based prevalence is clinically relevant, as asymptomatic ventricular preexcitation may be discovered incidentally and may require structured risk assessment and follow-up in pediatric cardiology settings. The prevalence of the WPW pattern observed in the present school-based screening cohort 0.074% is consistent with prior pediatric ECG screening literature. In a large Japanese school ECG screening program, Sano et al. reported a prevalence of ventricular preexcitation of approximately 0.07% in elementary and junior high school students, with a higher prevalence in high school students (≈0.17%), supporting an age-related increase in detection and a predominance of left-sided pathways [19]. Similarly, the epidemiologic overview summarized by Jung et al. described WPW prevalence estimates of approximately 0.07% in children aged 6–13 years and ≈0.17% in adolescents aged 14–15 years, further reinforcing that ventricular preexcitation may be more frequently identified with increasing age [20]. Longitudinal observational data from screening-detected WPW cohorts also suggest generally favorable outcomes; Inoue et al. followed 57 children with WPW detected during school heart screening over approximately 8 years and reported a benign overall clinical course, providing reassurance regarding conservative management strategies in selected cases [21]. In contrast to school-age cohorts, neonatal population screening in the Copenhagen Baby Heart Study identified the WPW pattern in 0.1% of 17,489 neonates, with a male predominance and primarily left-sided pathways, and with disappearance of the WPW pattern in most infants during follow-up, highlighting the dynamic nature of antegrade preexcitation across early development [7]. Taken together, these studies indicate that an asymptomatic WPW pattern prevalence in the range of 0.07–0.10% is typical in screened pediatric populations, placing the present results well within expected values while providing the first large-scale screening estimate from Romania.

The WPW pattern was more frequently observed in males than in females in this cohort; however, the difference was not statistically significant. Age-stratified prevalence varied between age groups, with the highest prevalence observed in 6-year-old children. These findings suggest that screening detection may vary across pediatric age strata and may be influenced by sampling distribution and the low absolute number of cases.

In the present cohort, the WPW pattern was observed more frequently in males than females (0.10% vs. 0.048%); however, this difference did not reach statistical significance, likely due to the small number of WPW cases. Nonetheless, the direction of effect is consistent with prior population-based and screening studies reporting a male predominance in ventricular preexcitation. In particular, the Copenhagen Baby Heart Study reported that approximately three-quarters of neonates with the WPW pattern were boys, supporting sex-related differences that may be apparent even early in life [7]. Similarly, large school-screening cohorts from Japan have also suggested a higher frequency of the WPW pattern in male students, although sex-specific prevalence estimates are not consistently reported across all studies. The biological basis of this sex imbalance remains uncertain, but may reflect developmental differences in atrioventricular conduction properties, accessory pathway persistence, or referral and detection patterns [19]. Taken together, these data suggest that while the WPW pattern remains uncommon in both sexes, screening programs may detect a modest male predominance, consistent with the trend observed in our cohort.

Age-related variation in WPW pattern prevalence was observed in our screening cohort, with the highest prevalence detected among 6-year-old children, followed by the 11–14 and 15–18 year strata, while the lowest prevalence was noted in the 7–10 year group. Although these differences should be interpreted cautiously due to the small absolute number of WPW cases, prior pediatric epidemiologic data support an age-dependent detection rate of ventricular preexcitation. In the Japanese school screening program reported by Sano et al., the prevalence of ventricular preexcitation was similar in younger school-age groups (~0.07%) but increased substantially in high school students (~0.17%), suggesting an increase in identification during adolescence [19]. Likewise, Jung et al. summarized population estimates showing an increase from approximately 0.07% in children aged 6–13 years to approximately 0.17% in adolescents aged 14–15 years, indicating that the WPW pattern may be detected more frequently with increasing age [20]. Several explanations may account for this trend, including developmental changes in conduction and fusion, increased ECG recording in older children (sports clearance or screening), and age-related differences in the persistence or penetrance of antegrade accessory pathway conduction. Overall, our findings are consistent with the prior literature suggesting that ventricular preexcitation prevalence is not constant across pediatric age groups and may increase during adolescence.

### 4.2. Phenotype of Preexcitation in Children

An important observation is that the majority of children demonstrated mild preexcitation, consistent with the recognized variability of ventricular preexcitation expression and the frequent incidental/asymptomatic detection of WPW pattern in the pediatric population [4]. In children, atrioventricular nodal conduction is typically rapid, and ventricular depolarization in WPW represents a fusion of AV nodal and accessory pathway conduction, such that the degree of manifest preexcitation may be limited despite the presence of an accessory pathway [3,22]. As a result, the PR interval may not appear markedly shortened, and the QRS complex may remain only mildly prolonged, increasing the likelihood of under-recognition of WPW pattern when strict adult ECG thresholds are applied [3,11]. This reinforces the importance of expert over-read in pediatric screening programs and supports the incorporation of subtle delta-wave morphology criteria and expanded ECG clues beyond the classical triad to improve detection of ventricular preexcitation in children [11,12].

Surface ECG-based algorithms suggested that accessory pathways were predominantly left-sided, with smaller proportions of right-sided and septal pathways. However, localization accuracy is limited when preexcitation is mild, as delta-wave amplitude and QRS morphology are less distinct. Nevertheless, broad categorization of pathways (left-sided vs. right-sided vs. septal) remains clinically useful, especially for anticipating procedural complexity and counseling families, while acknowledging that invasive electrophysiologic testing remains the definitive method for localization.

Secondary repolarization abnormalities are common in WPW due to altered ventricular activation. In this cohort, early repolarization was identified in one ECG, with inferior J-point elevation and notching/slurring. Early repolarization may coexist with pediatric WPW and may further complicate ST–T interpretation. This is particularly relevant in adolescents, in whom early repolarization is common and may resemble pathology in the presence of preexcitation-related ST–T changes. Mizumaki et al. [23] assessed 111 patients with the WPW pattern and found early repolarization in 48 (43%). By AP location, the left free wall had 35/75, the right free wall had 6/23, and the septal had 7/13 ER. ER was always observed in leads with positive deflection of the initial delta wave, suggesting a relationship between the initial activation vector and ER appearance. After ablation, ER persisted in 28 (25%), ER disappeared in 20 (18%), and ER newly developed in 8 (7%).

### 4.3. Automated ECG Interpretation: Clinical Implications in Screening

A key clinically relevant finding is that automated ECG interpretation frequently misclassifies WPW-associated ECG changes [13]. Specifically, automated algorithms generated false-positive diagnostic statements, including infarction/ischemia patterns, conduction defects (BBB/IVCD) [14], and axis deviation flags. Notably, none of the available automated interpretation statements explicitly identified WPW/preexcitation, despite confirmation by pediatric electrophysiologists.

This has direct clinical implications: pediatric screening ECGs are frequently interpreted initially by non-specialists, and computer-generated statements may drive clinical decision-making [24]. In the setting of WPW, repolarization and QRS abnormalities can mimic infarction, ischemia, or conduction disease, potentially leading to unnecessary anxiety, additional diagnostic testing (including emergency evaluation, troponin testing, echocardiography) [15], and inappropriate referrals. Conversely, failure to explicitly identify WPW can lead to delayed recognition and missed opportunities for structured risk stratification [16].

These findings support the need for either expert ECG over-read in screening settings or targeted improvements in pediatric ECG algorithm design, particularly for identifying subtle preexcitation patterns in children with rapid AV nodal conduction.

Importantly, the observed automated ECG interpretation errors should not be interpreted as device-specific limitations. Although the present study used a BTL CardioPoint–integrated interpretation system, similar rule-based and pattern-recognition approaches are employed across widely used commercial ECG algorithms. The difficulty in correctly identifying the WPW pattern reflects fundamental electrophysiological characteristics of ventricular preexcitation rather than a specific software deficiency. In the WPW pattern, ventricular activation represents a fusion between atrioventricular nodal and accessory pathway conduction, resulting in variable and sometimes subtle ECG expression. In pediatric populations, preexcitation may be particularly difficult to recognize, as PR interval shortening may be minimal, QRS duration may remain <120 ms, and delta waves may be subtle or intermittent. In addition, secondary repolarization abnormalities may mimic ischemia or conduction disturbances, leading to overlap with patterns commonly recognized by automated algorithms. These factors predispose misclassification across different ECG interpretation systems, and similar limitations have been reported in prior studies evaluating automated ECG analysis in both WPW and pediatric populations [13,14,16]. Collectively, these findings suggest that the observed errors reflect inherent challenges of automated ECG interpretation rather than limitations of a specific device.

### 4.4. Limitations

This study has several limitations. Firstly, the number of WPW cases was small, limiting statistical power for subgroup analyses. Secondly, accessory pathway localization was based on surface ECG algorithms without invasive electrophysiological confirmation. Thirdly, the study design was cross-sectional and based on screening ECG data, without the availability of longitudinal clinical follow-up, electrophysiological evaluation, or outcome data. Consequently, we were unable to perform formal risk stratification or assess the arrhythmic risk, natural history, or need for intervention in the identified cases. This is particularly relevant, as current management of asymptomatic WPW relies on risk stratification strategies (including ambulatory monitoring, exercise testing, and invasive electrophysiological study) to differentiate low-risk from high-risk accessory pathways. Therefore, the clinical applicability of our findings should be interpreted primarily in an epidemiological and screening context rather than a prognostic framework.

Fourthly, a formal assessment of inter-observer agreement in ECG interpretation was not performed. Although all suspected WPW cases were reviewed and adjudicated by experienced pediatric electrophysiologists, variability between observers was not quantified using standardized metrics such as kappa statistics. Therefore, a degree of inter-observer variability in the identification and classification of ventricular preexcitation cannot be excluded.

Fifthly, the extrapolation of WPW prevalence to the national level is subject to several limitations: the study population was derived from selected regions in northwest and west Romania and may not fully reflect geographic variability across the country; the screening program included only asymptomatic children without known cardiovascular disease, which may lead to underestimation of true prevalence in the general pediatric population; and the analysis assumes uniform prevalence across regions and demographic subgroups, which may not hold in practice. Therefore, the estimated national burden should be interpreted as an approximate, exploratory projection rather than a definitive population-level estimate.

## 5. Conclusions

In a large school-based ECG screening cohort of asymptomatic Romanian children aged 6–18 years, the WPW pattern was identified in 0.074%. Most cases exhibited mild preexcitation. Automated ECG interpretation frequently produced misleading diagnostic statements and did not reliably recognize preexcitation. These findings emphasize the importance of expert ECG review in pediatric screening programs and highlight the need for improved automated ECG algorithms in children with the WPW pattern.

## Figures and Tables

**Table 1:** Baseline characteristics of the screened cohort (*n* = 24,316).

Characteristic	Value
Total screened children, *n*	24,112
Age range, years	6–18
Female sex, *n* (%)	12,353 (50.8%)
Male sex, *n* (%)	11,963 (49.2%)
Age strata (*n*, %)	6 years: 186 (0.75%)
	7–10 years: 7790 (32.3%)
	11–14 years: 8829 (36.6%)
	15–18 years: 7307 (30.3%)

**Table 2:** Prevalence of WPW pattern in asymptomatic children (overall and by sex). Prevalence was calculated using the Wilson score method. Male vs. female: Fisher’s exact test *p* = 0.18.

Group	WPW Cases (*n*)	Total (*N*)	Prevalence (%)
Overall	18	24,112	0.074
Males	12	11,858	0.10
Females	6	12,255	0.048

**Table 3:** Age-stratified prevalence of WPW pattern. Highest prevalence observed in 6-year-old children, followed by 11–14 years and 15–18 years strata; lowest in 7–10 years group.

Age Category	WPW Cases (*n*)	Total in Age Category (*N*)	Prevalence (%)
6 years	1	186	0.53
7–10 years	2	7790	0.02
11–14 years	10	8829	0.11
15–18 years	5	7307	0.07

**Table 4:** ECG interpretation errors among WPW pattern ECGs (*n* = 18). All 18 ECGs were adjudicated as demonstrating ventricular preexcitation by pediatric electrophysiologists. ^1^ Failure to recognize WPW was defined as the absence of terms such as ‘preexcitation’, ‘WPW’, or “delta wave” in the automated statement.

Type of Automated ECG Interpretation Error	Definition (Examples)	Cases, *n* (%)
Infarction/ischemia false-positive	“possible myocardial infarction”, “ischemia”	1 (5.6%)
BBB/IVCD mislabel	RBBB/LBBB, nonspecific IVCD	5 (27.8%)
Axis deviation flagged	LAD or RAD statements	3 (16.7%)
Failure to recognize WPW pattern ^1^	WPW not explicitly stated despite EP adjudication	18 (100%)

## Data Availability

The data supporting reported results can be found archived in Mega Cloud and are available at the following link: https://mega.nz/fm/oJogGBJR (accessed on 21 January 2026).

## Abbreviations

The following abbreviations are used in this manuscript:

AP	Accessory pathway
APERP	Accessory pathway effective refractory period
AF	Atrial fibrillation
WPW	Wolff–Parkinson–White
